# Hepatic Estrogen Receptor Alpha Overexpression Protects Against Hepatic Insulin Resistance and MASLD

**DOI:** 10.3390/pathophysiology32010001

**Published:** 2025-01-03

**Authors:** Ester S. Alves, Jessica D. M. Santos, Alessandra G. Cruz, Felipe N. Camargo, Carlos H. Z. Talarico, Anne R. M. Santos, Carlos A. A. Silva, Henrique J. N. Morgan, Sandro L. Matos, Layanne C. C. Araujo, João Paulo Camporez

**Affiliations:** 1 Department of Physiology, Ribeirão Preto School of Medicine, University of São Paulo, Ribeirão Preto 14049-900, Brazil; alvesssester@gmail.com (E.S.A.); jessicadenielle@usp.br (J.D.M.S.); alessandracruz@usp.br (A.G.C.); felipe_camargo@usp.br (F.N.C.); carlos.talarico@usp.br (C.H.Z.T.); annemello1928@hotmail.com (A.R.M.S.); caguiar@usp.br (C.A.A.S.); morgan.hjn@usp.br (H.J.N.M.); sandroleaomatos@gmail.com (S.L.M.); 2 Superior Institute of Biomedical Sciences, State University of Ceara, Fortaleza 60714-903, Brazil; layanne.araujo@uece.br

**Keywords:** insulin action, estrogen signaling, hepatic insulin resistance, MASLD

## Abstract

**Background/Objectives:** Metabolic dysfunction-associated steatotic liver disease (MASLD) is associated with cardiometabolic risk. Although studies have shown that estradiol positively contributes to energy metabolism via estrogen receptor alpha (ERα), its role specifically in the liver is not defined. Therefore, this study aimed to evaluate the effects of ERα overexpression, specifically in the liver in mice fed a high-fat diet (HFD). **Methods:** Male C57BL/6J mice were divided into four groups, vehicle fed with regular chow (RC) (RC-Vehicle); vehicle fed an HFD (HFD-Vehicle); AAV-treated fed with RC (RC-AAV); and AAV-treated fed an HFD (HFD-AAV), for 6 weeks (8–10 mice per group). AAV was administered intravenously to induce ERα overexpression. **Results:** We demonstrate that overexpression of ERα in RC-fed mice reduces body fat (28%). These mice show increased oxygen consumption in cultured primary hepatocytes, both in basal (19%) and maximal respiration (34%). In HFD-fed mice, we showed a decrease in hepatic TAG content (43%) associated with improved hepatic insulin sensitivity (145%). **Conclusions:** From this perspective, our results prove that hepatic ERα signaling is responsible for some of the metabolic protective effects of estrogen in mice. Overexpression of ERα improves hepatocyte mitochondrial function, consequently reducing hepatic lipid accumulation and protecting animals from hepatic steatosis and hepatic insulin resistance. Further investigations will be needed to determine the exact molecular mechanism by which ERα improves hepatic metabolic health.

## 1. Introduction

Lifestyles, such as a sedentary lifestyle and high-calorie consumption, contribute significantly to the development of conditions such as type 2 diabetes mellitus (T2DM), obesity, insulin resistance, and cardiovascular diseases [1], which together are called metabolic syndrome, with insulin resistance associated with obesity being the main factor in this syndrome. Several mechanisms are believed to be responsible for insulin resistance, including abnormal lipid metabolism and ectopic lipid accumulation [2], mitochondrial dysfunction [3], and inflammation [4].

These factors also contribute to the development of metabolic dysfunction-associated steatotic liver disease (MASLD), which begins with the accumulation of triglycerides (TAG) in the liver and is characterized by the presence of lipid droplets in more than 5% of hepatocytes [5]. Recent studies have shown that 38% of adults around the world have MASLD, and this prevalence is expected to rise to over 50% by 2040 [6,7]. Hepatic insulin resistance has been closely associated with increased ectopic lipids accumulation in the liver, i.e., it is correlated with MASLD. High-fat diet in mice and rats is associated with increased hepatic steatosis and increased insulin resistance in the liver [8,9,10,11]. The mechanism suggested for the hepatic steatosis related to insulin resistance is that lipid intermediates, especially DAG, can activate PKC, which binds to IR and reduces its activity, consequently reducing IRS phosphorylation and PI3-K/Akt signaling [9], which leads to reduced insulin-stimulated hepatic glycogen synthesis and increased endogenous glucose production [12].

This relationship between increased hepatic steatosis and insulin resistance in the liver is also present in humans. High-fat consumption has been shown to increase liver fat content, whereas low-fat diets reduce human liver fat content [13]. Furthermore, obese humans with MASLD showed insulin resistance associated with increased DAG content and PKCε activation [14]. All this demonstrates the critical pathophysiological role of hepatic fat accumulation observed mainly in obesity, leading to the development of MASLD and hepatic insulin resistance.

Studies show differences in the development of MASLD between men and women. Women of reproductive age have a lower risk of advanced fibrosis compared to men of the same age group [15]. This protection in women is due to the presence of the hormone estradiol (E2), which plays a crucial role in glucose homeostasis [16]. Experimental studies show that E2 regulates body weight, fat, and insulin sensitivity [17]. E2 treatment in ovariectomized female mice fed an HFD improves body composition and increases insulin sensitivity by reducing ectopic lipid content in the liver and muscle [17].

Furthermore, it has been demonstrated that the beneficial effects of E2 are associated with the activation of estrogen receptor alpha (ERα). Activation of ERα increases mitochondrial activity and protects against high-fat diet-induced obesity in ovariectomized female mice [18]. In the liver, lack of ERα is associated with increased TAG deposition and increased susceptibility to high-fat diet-induced MASLD. In contrast, activation of ERα by tamoxifen improves metabolism in rodents by increasing GDF15 secretion [19]. Studies also show that ERα expression is significantly lower in males than in females in mice and pigs, and this reduction is associated with lower glucose tolerance in males [20]. However, no study has evaluated the specific increase in ERα, particularly in the liver, allowing for us to study the effects of ERα signaling without the global effects of E2. Knowing that E2 exerts beneficial effects on energy metabolism via ERα and that females are physiologically protected against lipid-induced insulin resistance when compared to males, our objective is to evaluate in vivo the impact of ERα overexpression, specifically in the liver on the hepatic energy metabolism and hepatic insulin resistance in male mice.

## 2. Materials and Methods

### 2.1. Animals

Eight-week-old male mice (C57BL/6J) (~22 g) were used for the experiments. They were maintained in a room with controlled temperature at 22 ± 2 °C and had ad libitum access to food and water. This room had a 12-h light/dark cycle. The AAV-Esr1 expression plasmid was created by cloning a mouse ERS1 full-length DNA into an AAV8-TBG vector (Vector BioLabs, Malvern, PA USA). Viruses were amplified, packaged, and purified by Vector BioLabs. Mice at 8 weeks old were tail-vein injected with AAV8-TBG-m-Esr1 at 1 × 10^10^ gc/mouse in 150 μL of PBS (Vector BioLabs, Malvern, PA, USA). The viral dose was based on a previously published report [21]. This AAV was used to promote the overexpression of ERα specifically in the liver, or with the vehicle used to dilute the viral solution. After that, mice were fed with regular chow (RC) or a high-fat diet (HFD) containing 45% of calories from fat (D12451, Research Diets, NJ, USA) for 6 weeks. They were divided into four groups (*n* = 8): vehicle fed with RC (RC-Vehicle); vehicle fed with HFD (HFD-Vehicle); AAV fed with RC (RC-AAV); AAV fed with HFD (HFD-AAV). The Bruker Minispec analyzer (Bruker BioSpin, Billerica, MA, USA) was used to assess mice body composition. All experiments carried out were previously approved in accordance with the guidelines of the Ethics Committee of Ribeirão Preto School of Medicine of the University of São Paulo, with protocol 084/2021.

### 2.2. Hyperinsulinemic–Euglycemic Clamp Studies

In order to evaluate insulin sensitivity, a hyperinsulinemic–euglycemic clamp was performed as previously described [17]. Briefly, a jugular vein catheter was implanted in mice 5–7 days before the experiments. In order to assess basal glucose metabolism, [3-^3^H]-glucose (Perkin-Elmer Life Sciences, Hopkinton, MA, USA) was intravenously infused at a rate of 0.05 μCi/min for 120 min after a period of 6 h of fasting. After that, the hyperinsulinemic–euglycemic clamp was applied in awake animals for 120 min with a primed insulin infusion for 3 min (29 mU/kg); following the prime period, we performed a continuous infusion (3 mU/kg/min) of regular insulin (Novolin; Novo Nordisk, Bagsværd, Denmark). A continuous infusion of [3-^3^H]-glucose (0.1 μCi/min) was administered in order to measure whole-body glucose turnover. In order to maintain the target blood glucose (~120 mg/dL), a variable infusion of dextrose (20%) was used. The animals were anesthetized at the end of the clamp with an injection of sodium pentobarbital (150 mg/kg). Liver, muscle, and adipose tissue were removed and immediately frozen in liquid nitrogen. They were stored at −80 °C for subsequent analyses.

### 2.3. Glucose Tolerance Test

Glucose tolerance tests (GTTs) were conducted after 6 hours of fasting. After a basal blood sample was collected, mice were intraperitoneally injected with glucose at the dose of 1 mg/kg body weight (10% dextrose). Glucose was determined from blood samples collected by tail bleeding at 0, 15, 30, 45, 60, 90, and 120 min after the injection. The blood glucose was measured with portable commercial glucometer.

### 2.4. Tissue Lipid Content and Histological Analysis of Oil Red

Liver was collected from all animals after 6 hours fasting. This tissue was used to quantify hepatic triglycerides (TAG), using the Bligh and Dyer method [22] and measured using TAG reagent (Bioclin, Belo Horizonte, Brazil). Liver fragments were used for ORO staining. The images were obtained using a Nikon Eclipse Ti-U microscope with a 20X objective, a Nikon DS-Ri1 digital camera, and NISElements BR 3.1 software. The fat accumulation in the tissue was quantified using the Image J program (version 1.54).

### 2.5. Western Blotting

In order to evaluate protein content in liver, Westen blot was performed in pieces of the liver (~50 mg) homogenized in RIPA buffer with protease and phosphatase inhibitor cocktail (TermoFisher, Waltham, MA, USA). The membranes were incubated with the following antibodies: ERα e VDAC (Cell Signaling Technology, Inc., Danvers, MA, USA). All membranes were incubated with GAPDH (Santa Cruz Biotechnology, Inc., Santa Cruz, CA, USA) to control the amount of protein in the membrane.

### 2.6. Assessment of Gene Expression—Polymerase Chain Reaction (PCR)

RNA was extracted from pieces of liver samples in Trizol (Life Technologies, Carlsbad, CA, USA). Then, RNA was used for cDNA preparation through reverse transcription reaction (High-Capacityc DNA kit, Applied Biosystems, Waltham, MA, USA). Specific gene expression was evaluated by real-time PCR (Rotor Gene Q—Qiagen) and SYBR Green fluorescent probe (Platinum^®^ SYBR^®^ Green qPCR Supermix UDG, Invitrogen, Waltham, MA, USA). Gene expression analyses were performed using a method described by Livak and Schmittgen [23] and Pfaffl [24]. The genes analyzed were Esr1 (Estrogen Receptor 1) and GAPDH (housekeeping).

### 2.7. Measuring Oxygen Consumption in Primary Hepatocytes

Primary hepatocytes from mice treated with saline or AAV were isolated. After the cells were washed with recovery medium (DMEM with high glucose plus 10% fetal bovine serum), they were seeded into each well of a Seahorse XF96 cell culture plate (5000) (Seahorse Bioscience, North Billerica, MA, USA). The cells were kept in recovery medium for 4 h and then washed with DMEM medium (low glucose plus 10% fetal bovine serum) and cultured overnight. Then, the cells were washed with pre-warmed XF96 assay medium (~37 °C) and the XF96 assay medium (180 μL) was added. Immediately before the measurements, the cells with assay medium were placed in a humidified incubator without buffer at 37 °C for 1 h to allow for temperature and pH equilibrium. XF Assay medium (200 μL) was added, and the samples were analyzed on the XF24 analyzer.

### 2.8. Statistics 

The results were analyzed using GraphPad Prism version 9.0 (GraphPad Software, La Jolla, CA, USA) using two-way analysis of variance (ANOVA) followed by Bonferroni’s test for homogeneity of variances. When necessary, T test for unpaired samples was used. The results are expressed as means ± SEM. The minimum acceptable significance level was *p* < 0.05.

## 3. Results

### 3.1. ERα Overexpression Improves Body Composition

**Figure 1:**
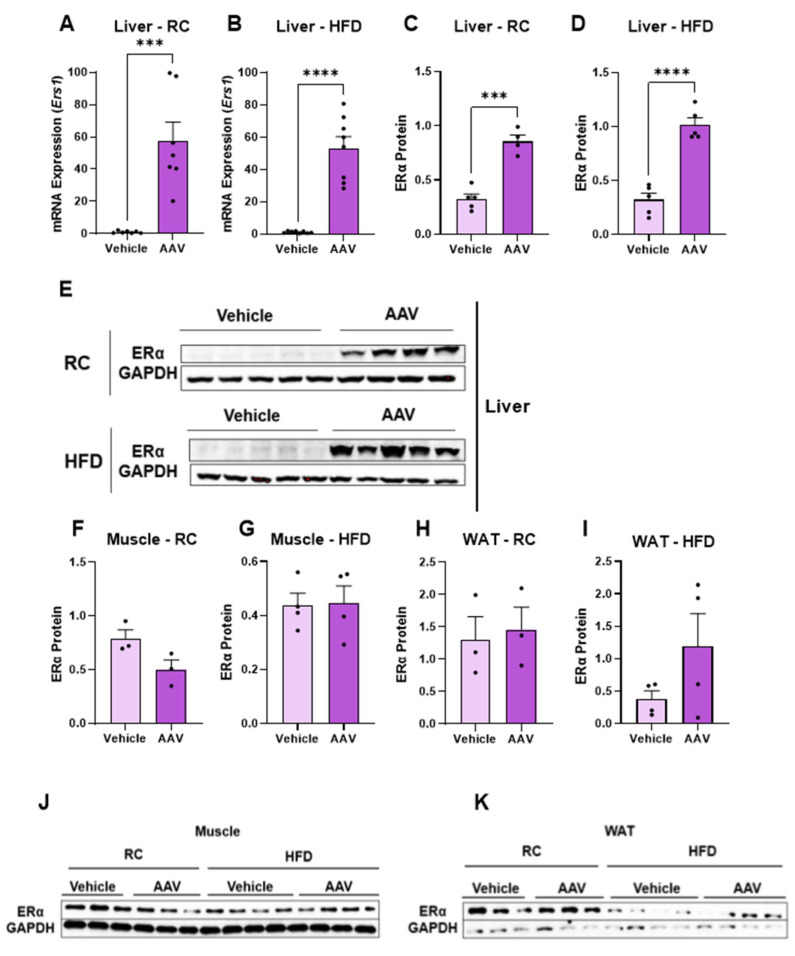
AAV administration increases ERα expression in the liver and not in other tissues. Analysis of ERα overexpression in different tissues using RT-qPCR and Western blotting. (**A**) Esr1 mRNA expression in the liver of RC-fed mice. (**B**) Esr1 mRNA expression in the liver of mice fed HFD. (**C**) ERα protein expression in the liver of mice fed RC. (**D**) ERα protein expression in the liver of mice fed HFD. (**E**) ERα protein qualitative analysis in mice fed RC and HFD, using ImageJ. (**F**) ERα protein expression in the muscle of mice fed RC. (**G**) ERα protein expression in the muscle of mice fed HFD. (**H**) ERα protein expression in the brown adipose tissue of mice fed RC. (**I**) ERα protein expression in the brown adipose tissue of mice fed HFD. (**J**) ERα protein qualitative analysis in the muscle of mice fed RC and HFD, using ImageJ. (**K**) ERα protein qualitative analysis in WAT in mice fed RC and HFD, using ImageJ. Data are represented as means ± SEM (*n* = 5–10), with differences considered significant when *p* < 0.05. In A–D and F–I analysis, *T* test was performed, significance level *p* < 0.05. (*** *p* < 0.001); (**** *p* < 0.0001).

In order to evaluate if the AAV was efficient and specific to modulate Ers1 gene expression and ERα protein content, RT-PCR and Western blot were performed in liver, muscle, and white adipose tissue. The expression of the Esr1 (Estrogen Receptor 1) gene in the liver of the mice that received the AAV was increased compared to those that only received the vehicle (Figure 1A,B). The expression of the ERα protein was also increased (Figure 1C,D) in those fed RC or an HFD. Muscle and white adipose tissue (WAT) were also analyzed, showing no significant difference in protein expression, thus confirming overexpression in specific tissue (Figure 1F–I).

**Figure 2:**
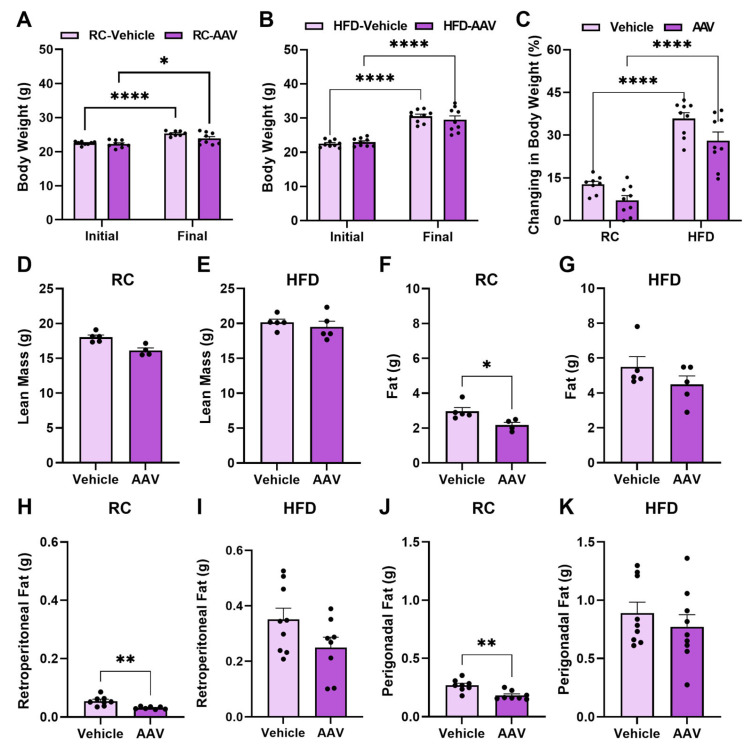
Overexpression of ERα in the liver improves the body composition of mice fed RC. Analysis of the body composition of mice fed RC and HFD. (**A**) Comparative analysis of weight before and after virus inoculation in mice fed RC. (**B**) Comparative analysis of weight before and after virus inoculation in mice fed an HFD. (**C**) Percentual changing in body weight of mice. (**D**) Lean mass of mice fed RC. (**E**) Lean mass of mice fed HFD. (**F**) Total fat of mice fed RC. (**G**) Total fat of mice fed HFD. (**H**) Retroperitoneal fat through magnetic resonance imaging of RC-fed mice. (**I**) Retroperitoneal fat through magnetic resonance imaging of mice fed HFD. (**J**) Perigonadal fat through magnetic resonance imaging of RC-fed mice. (**K**) Perigonadal fat on MRI scans of mice fed HFD. Data are represented as means ± SEM (*n* = 5–10), with differences considered significant when *p* < 0.05. In A and B, a two-way ANOVA with Bonferroni’s post-hoc was performed. For C–J analysis, *T* test was performed, significance level *p* < 0.05. (* *p* < 0.05); (** *p* < 0.01); (**** *p* < 0.0001).

Regarding body weight, we identified no significant differences between vehicle and AAV (Figure 2A,B). The lean mass showed no significant difference between the groups (Figure 2C,D). However, mice fed RC with ERα overexpression showed reduced total, retroperitoneal, and perigonadal fat content when compared to controls (Figure 2E,G,I), decreasing by approximately 26%, 42%, and 32%, respectively.

### 3.2. ERα Overexpression Improves Hepatic Insulin Sensitivity

**Figure 3:**
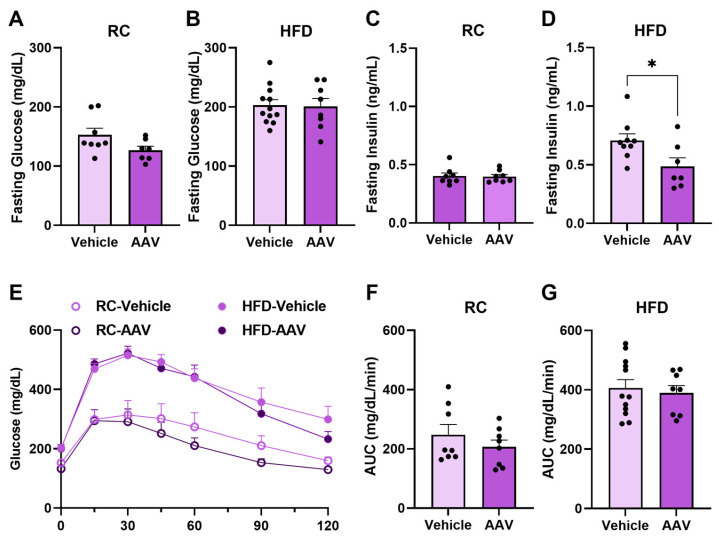
Overexpression of ERα is involved in glucose metabolism in mice fed an HFD. Evaluation of glucose metabolism using ipGTT. (**A**) Fasting glycemia of mice fed RC. (**B**) Fasting glycemia of mice fed an HFD. (**C**) Fasting insulin in mice fed RC. (**D**) Fasting insulin in mice fed an HFD. (**E**) GTT curve analysis. (**F**) Area under the curve of mice fed RC. (**G**) Area under the curve of mice fed HFD. Data are represented as means ± SEM (*n* = 5–10), with differences considered significant when *p* < 0.05. In A–D, F, and G analysis, *T* test was performed, significance level *p* < 0.05. For E, a two-way ANOVA with Bonferroni’s post-hoc was performed. (* *p* < 0.05).

We assessed fasting blood glucose and insulin concentrations. We observed no significant differences between RC-fed mice. However, we did show that the group with ERα overexpression, fed an HFD, showed a reduction in fasting insulin (*p* < 0.0180) (Figure 3D). When the ipGTT curve and the area under the curve (AUC) were analyzed, no significant differences were found between the groups’ vehicle vs. AAV (Figure 3E–G). However, the HFD-fed mice displayed increased glucose intolerance compared to RC-fed mice.

**Figure 4:**
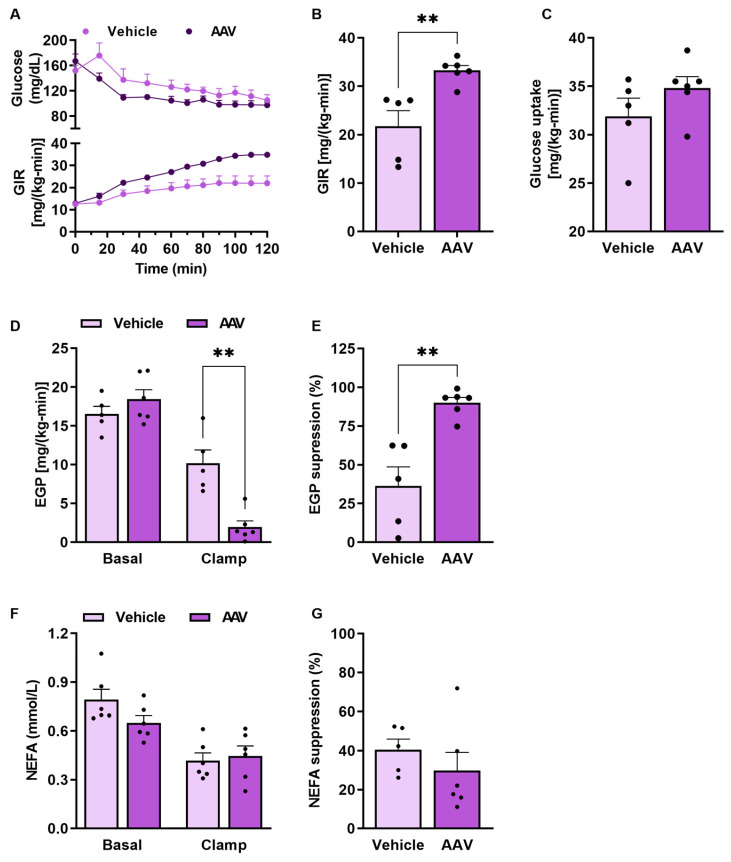
ERα overexpression regulates insulin sensitivity. (**A**) The glucose infusion rate (GIR) and plasma glucose curve during clamp. (**B**) Average of GIR during the last 40 min of the clamp. (**C**) Whole-body glucose disappearance during clamp. (**D**) Basal endogenous glucose production (EGP) and clamp EGP. (**E**) Suppression percentage of EGP. (**F**) Basal and clamp non-esterified fatty acids (NEFAs). (**G**) Insulin-stimulated NEFA suppression during the clamp. Data are represented as means ± SEM (*n* = 5–10), with differences considered significant when *p* < 0.05. In A and F, a two-way ANOVA with Bonferroni’s post-hoc was performed. For B–E and G analysis, *T* test was performed, significance level *p* < 0.05. (** *p* < 0.01).

To evaluate insulin sensitivity, we performed the hyperinsulinemic–euglycemic clamp in HFD-fed mice. Hepatic ERα overexpression markedly improved whole-body insulin sensitivity, as demonstrated by the increase in glucose infusion rate in AAV mice (Figure 4A,B). However, there was no difference in whole-body glucose uptake (Figure 4C). Although there was no difference in basal endogenous glucose production (EGP) between the groups (Figure 4D), EGP suppression was higher in ERα overexpression compared to controls (Figure 4D,E), indicating greater hepatic insulin sensitivity. Also, there was no difference in non-esterified fatty acid (NEFA) concentrations between the groups either in fasting and during the hyperinsulinemic–euglycemic clamp (Figure 4F,G).

### 3.3. ERα Overexpression Regulates Hepatic Lipid Content

**Figure 5:**
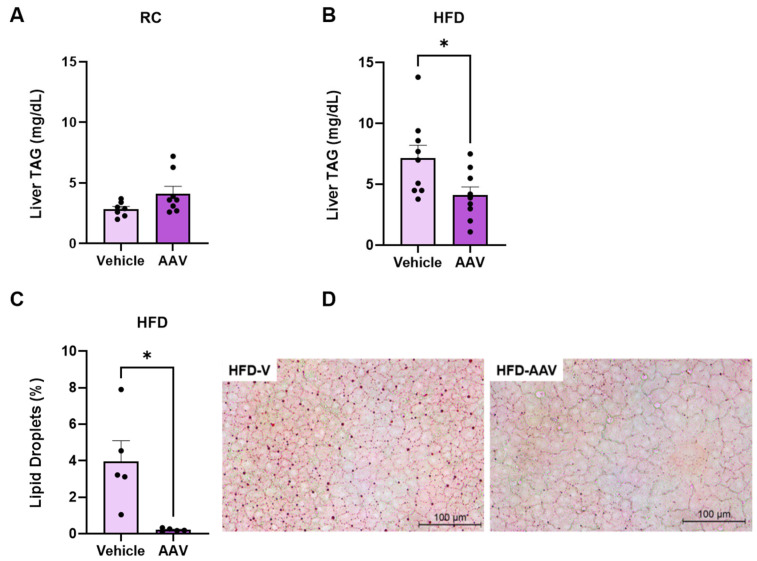
ERα overexpression regulates hepatic lipid content. Analysis of quantitative TAG dosage in the liver of mice fed RC and an HFD. (**A**) TAG content in the liver of mice fed RC. (**B**) TAG content in the liver of mice fed an HFD. (**C**) Quantitative analysis of lipid droplets by Oil Red O in mice fed an HFD. (**D**) Representative image of the liver, stained with Oil Red O. Data are represented as means ± SEM (*n* = 5–10), with differences considered significant when *p* < 0.05. In A, B, and C analysis, T test was performed, significance level *p* < 0.05. (* *p* < 0.05).

In order to assess if the improved hepatic insulin sensitivity in AAV-treated mice was associated with ectopic lipid content, hepatic TAG was assessed in these mice. TAG levels in liver tissue showed significant differences in the group fed HFD with ERα overexpression (Figure 5A,B). We also found a reduction of approximately 95% in lipid droplets in the liver tissue of HFD-fed mice with ERα overexpression (Figure 5C,D) compared to vehicle animals.

### 3.4. Overexpression of ERα Increases Cellular Respiratory Capacity

**Figure 6:**
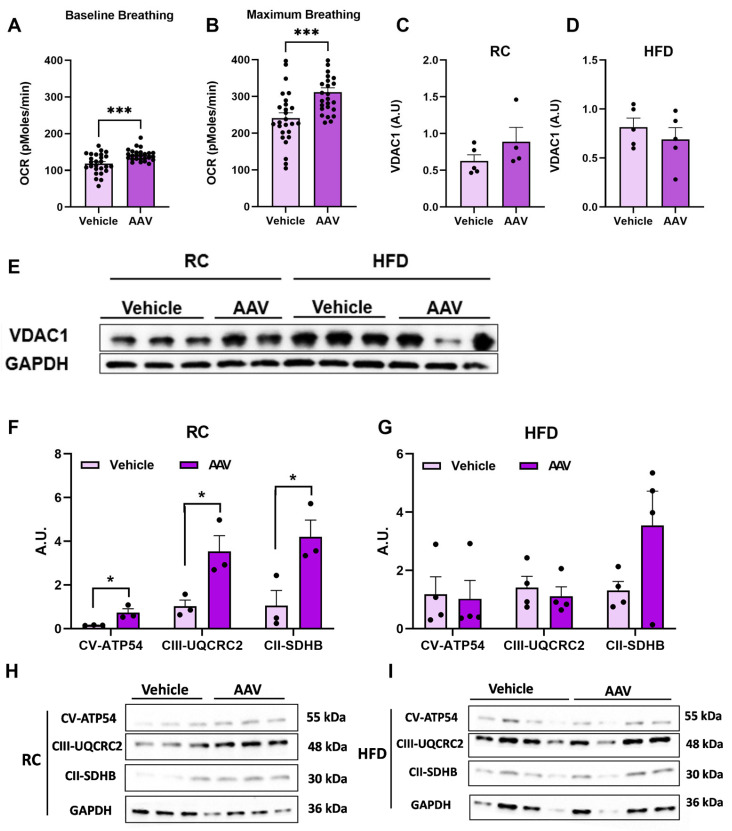
Overexpression of ERα increases cellular respiratory capacity. Analysis of mitochondrial function in animals fed RC and an HFD. (**A**) Basal O2 consumption rates (OCR) in isolated hepatocytes. (**B**) Maximum O2 consumption rates (OCR) in isolated hepatocytes. (**C**) VDAC1 protein expression in mice fed RC. (**D**) VDAC1 protein expression in mice fed an HFD. (**E**) VDAC1 protein qualitative analysis using ImageJ. (**F**) Total OXPHOS protein expression in mice fed RC. (**G**) Total OXPHOS protein expression in mice fed an HFD. (**H**) Total OXPHOS protein qualitative images from mice fed RC. (**I**) Total OXPHOS protein qualitative images from mice fed an HFD. Data are represented as means ± SEM (*n* = 5–10), with differences considered significant when *p* < 0.05. In A–B and D–E analysis *T* test was performed, significance level *p* < 0.05. (* *p* < 0.05); (*** *p* < 0.001).

We also assessed oxygen consumption in isolated hepatocytes and evidenced an increase of approximately 19% in the basal respiration rate in hepatocytes isolated from mice overexpressing ERα (Figure 6A) (Table 1). This increased oxygen consumption was also followed by an increase in maximal respiratory capacity by ~34% (Figure 6B) (Table 1). To evaluate if this increased O2 consumption in hepatocytes was due to increased mitochondrial density, we analyzed the Voltage-Dependent Anion Channel (VDAC) protein, and there was no significant difference observed between the groups (Figure 6C–E). However, when total OXPHOS protein content was analyzed, we observed increased expression in AAV mice fed RC compared with vehicle (Figure 6F), with no difference between groups in mice fed an HFD (Figure 6G).

## 4. Discussion

The objective of this work was to evaluate in vivo the effects of ERα overexpression, specifically in the liver, on hepatic energy metabolism and hepatic insulin resistance in mice. In general, it was observed that animals with overexpression of ERα in the liver showed protection against hepatic steatosis induced by a high-fat diet, associated with improved liver insulin sensitivity. These results can be explained, at least partially, by increased mitochondrial respiration in hepatocytes.

It has been consistently shown that the E2 action plays a vital role in metabolic control [8,17,25,26]. Studies suggest that hepatic E2 signaling may also limit these effects in men [25,27,28]. From this perspective, it was demonstrated that male ERα-liver knockout (LKO) mice presented insulin resistance, associated with an increase in proteins involved in the synthesis of fatty acids and an increase in the accumulation of TAG in the liver, a decrease in hepatic glycogen synthesis, and an increase in of hepatic glucose production during hyperinsulinemia [28]. Our data corroborate these studies, as animals with increased ERα signaling in the liver showed protection against TAG accumulation and improved hepatic insulin resistance. Thus, this evidence suggests that hepatic ERα signaling may mediate the protective metabolic effects of E2.

In this study, we demonstrated that mice overexpressing ERα suppressed endogenous glucose production more than controls, indicating greater hepatic insulin sensitivity, which was associated with a reduction in hepatic TAG content. Our data corroborate previously observed results, which showed that ovariectomized female mice present increased insulin resistance and hepatic steatosis, while intact females present protection against these effects of the high-fat diet when compared to males. In line, it has been shown that male LKO mice develop insulin resistance and impaired insulin signaling in the liver [28]. Experimental studies have demonstrated that consuming a high-fat diet results in hepatic steatosis and hepatic insulin resistance, associating these two phenomena as cause and effect [28]. Generally, this increase in hepatic TAG content is related to a rise in other signaling lipid species (DAG and ceramides) that directly cause a reduction in insulin signaling [29], which thus leads to hepatic insulin resistance. Our animals with ERα overexpression in the liver showed a decrease in hepatic TAG content of ~40%, associated with improving hepatic insulin sensitivity. This improvement may be due to the reduction in hepatic DAG, as previously observed in models related to E2 action [30] or models associated with genetic or dietary manipulation [29,30].

The reduction in hepatic steatosis observed in animals with ERα overexpression in the liver may be due to an increase in mitochondrial function, as we observed increased oxygen consumption in the hepatocytes of these animals. It has already been observed in other animal models that changes in mitochondrial function in hepatocytes lead to modulation of hepatic TAG content [30,31]. Therefore, increased oxygen consumption in these cells could lead to a better ability of hepatocytes to deal with the excess energy that arrives when feeding rodents a high-fat diet. Interestingly, it has been shown that E2 can modulate mitochondrial function. It was previously observed that reducing ERα expression in myocytes led to a marked reduction in mitochondrial functionality, including reduced oxygen consumption rates caused by diminished mtDNA turnover due to a balanced decrease in mtDNA replication and degradation [30]. Meanwhile, OVX mice treated with E2 showed greater oxygen consumption by hepatocytes. Interestingly, this increase in mitochondrial function observed in our study was apparently due to a rise in total mitochondrial content observed by increased OXPHOS protein content in AAV mice. In further studies, it would be interesting to determine how ERα affects the functionality and content of mitochondria in hepatocytes.

Taken together, our data demonstrated that overexpression of ERα, specifically in the liver, improves mitochondrial function, reducing lipid accumulation and thus protecting animals from hepatic steatosis and hepatic insulin resistance. Therefore, we believe our study will help expand on existing knowledge regarding the mechanisms of ERα signaling and its effects by providing evidence that hepatic ERα signaling has a beneficial impact on liver energy metabolism and hepatic insulin resistance.

## Figures and Tables

**Table 1:** Mitochondrial stress test performed in primary hepatocytes.

	Vehicle	AAV
Baseline	118 ± 5.3	141 ± 3.1 ***
Oligomycin	107 ± 5.2	112 ± 3.3
FCCP	235 ± 13.4	315 ± 11.6 ***
Rotenone and antimycin A	114 ± 7.4	106 ± 5.3

*** represents *p* < 0.001 compared with vehicle.

## Data Availability

The raw data supporting the conclusions of this article will be made available by the authors on request.
